# The unequal impact of Covid‐19 on the lives and rights of the children of modern slavery survivors, children in exploitation and children at risk of entering exploitation

**DOI:** 10.1111/chso.12572

**Published:** 2022-04-21

**Authors:** Erika Jiménez, Vicky Brotherton, Alison Gardner, Nicola Wright, Hannah Browne, Nancy Esiovwa, Minh Dang, Emily Wyman, Liana Bravo‐Balsa, Benjamin Lucas, Mohsen Gul, Elizabeth Such, Zoe Trodd

**Affiliations:** ^1^ University of Nottingham UK; ^2^ Bradford Survivor Alliance UK; ^3^ The University of Sheffield UK; ^4^ Present address: Queen's University UK

**Keywords:** child protection, child trafficking, children's rights, Covid‐19, modern slavery

## Abstract

This article discusses the unequal impact of Covid‐19 on the lives of the children of survivors of modern slavery, child victims of exploitation and children at risk of exploitation in the UK. It draws on research that has analysed the risks and impacts of Covid‐19 on victims and survivors of modern slavery. It explores how pandemic responses may have hindered these children's rights to education, food, safety, development and participation and representation in legal processes. It suggests that the pandemic should be used as an impetus to address inequalities that existed pre‐Covid‐19 and those that have been exacerbated by it.

## INTRODUCTION

Pandemic policy responses in the UK may have contributed to limiting the spread of Covid‐19 (Department of Health and Social Care, 2020), however, certain already marginalised groups have been side‐lined such as children and young people. Recent research on Covid‐19 and children shows that the pandemic has been profoundly experienced by children worldwide (Aitken, 2021; Bessell, 2021; Cortés‐Morales & Morales, 2021; Million, 2021). Highlighted in this literature in the fields of children's rights, children's geographies and social studies of childhood, is the way in which the crisis has shed light on and exacerbated pre‐existing inequalities both globally and locally. It has also ‘invisibilised’ children in the media, public spaces and policy debates (Cortés‐Morales et al., 2021) and children's voices have not been consulted in matters which have severely affected them (Bessell, 2021; Lundy et al., 2021; Million, 2021).

In the UK, as in other countries, these adverse impacts on children have not only been significant, but also differentiated across the country and the shocks have been unequally felt by children especially those experiencing poverty, migrant and asylum seeking children as well as those with disabilities (Lundy et al., 2021; Lynch & Kilkelly, 2021). As other pandemics such as Ebola, Zika, HIV/AIDS, SARS and H1N1 have demonstrated, the ensuing economic instability, quarantines, school closures and issues accessing healthcare can threaten human rights and facilitate the enslavement of people (Peterman et al., 2020). Whilst studies have reported the impact of the pandemic on marginalised children around the world, few have focused specifically on children affected by modern slavery either as the children of those who have exited situations of slavery, child victims of exploitation or those at risk of entering exploitation. Research has been undertaken in relation to the implications of the crisis for child criminal exploitation in the UK (Brewster et al., 2021; Pitts, 2020; Racher & Brodie, 2020). This emerging research indicates that the safeguarding ability of organisations in England was hindered by the Covid‐19 restrictions especially, the reduction in face‐to‐face contact with young people exploited for County Lines purposes (Brewster et al., 2021). This paper seeks to contribute to this new body of evidence by exploring how lockdowns, school closures and the migration from in‐person to remote forms of support and provision specifically impacted the rights and lives of children of modern slavery survivors, unaccompanied asylum seeking children (UASC hereafter) as well as victims of criminal exploitation. This paper also indicates that there may be additional risks of exploitation facing children in the UK.

This article is informed by research which explored the potential risks and known impacts that Covid‐19 posed for victims and survivors of modern slavery and the mitigation strategies that had been implemented for this population at national, local and individual organisations levels. Specifically, it draws on a rapid evidence review, discussions held at a dialogue event with the UK anti‐slavery sector and interviews with (adult) survivors.

### Pre‐existing inequalities experienced by children in the UK

Although death rates resulting from Covid‐19 have significantly fallen (at the time of writing this article), there have been around 162 582 in the UK who have lost their lives to the disease (Dong et al., 2020). The UK became one of the epicentres of the virus in the Global North arguably because the UK Government was slow to respond at the initial stages of the pandemic compared to other neighbouring countries. After the Prime Minister's initial reluctance to act, the UK entered a national lockdown on 23 March 2020. In the UK, devolved administrations are responsible for policies relating to issues such as health and social care, housing and education (Department for Levelling Up et al., 2020). Therefore, in May 2020, the four nations of the UK (England, Northern Ireland, Scotland and Wales) began to diverge in their Covid‐19 containment and closure policies, with Scotland adopting the most stringent policies and England the least (Cameron‐Blake et al., 2020). It is also important to acknowledge the larger context within which children in the UK live and in which the pandemic took place.

At the time that the World Health Organization (WHO hereafter) declared SARS‐CoV‐2 a global pandemic, the UK had just begun the process of exiting the European Union which had brought about uncertainty and the threat of economic instability. Prior to Brexit and leading up to the pandemic, the UK had also experienced a decade of austerity policies which had led to underfunded social and children's services, healthcare and education (Blackburn, 2020; Crawford, 2020) despite promises that these would be ringfenced (Davies & Sloman, 2020; Kmietowicz, 2014). Therefore, although the UK provides free healthcare, the National Health Service (NHS) had been under‐resourced for years leading up to the pandemic which made it more vulnerable when the coronavirus outbreak occurred. Furthermore, cuts to social care and early years child support services such as the closure of SureStart centres also reduced the support available to families (Bate & Foster, 2017). In 2015, the UK Government also abolished its child poverty target a week before making cuts to welfare (Torjesen, 2015).

Austerity policies have also negatively impacted children's right to education, despite claims that it too would be ringfenced (Davies & Sloman, 2020). According to a report by the Institute for Fiscal Studies, funding for state schools in England experienced its most significant fall since the 1980s from 2010 following the forming of the Conservative‐led coalition (Belfield & Sibieta, 2016). Before the pandemic, children from lower‐income families experienced an attainment gap which was often exacerbated during school holidays. Contributing to the attainment gap is digital exclusion and poverty. As Watts (2020) comments, digital exclusion has implications for numerous human rights such as access to adequate healthcare, information and education as well as having consequences for mental health and well‐being. Austerity measures and the restructuring of the welfare system since 2012 in the UK also had a detrimental effect on children's right to food (Raj, 2019). Prior to the pandemic, around nine out of 30 pupils in a UK classroom experienced poverty (Penington, 2020). As with the attainment gap and digital inequalities, food insecurity amongst children rose as a result of school closures, unemployment and the economic shock of the pandemic (The Food Foundation, 2021a). This crisis, in part, led Manchester United footballer Marcus Rashford's campaign for Free School Meals to be available over the summer holidays and to go on to form the End Child Food Poverty Task Force.

This brief overview of the UK context illustrates how inequalities existed before the pandemic and how marginalised children are often the ones who bear the brunt of economic shocks, austerity policies and underfunded education and healthcare and yet, they have been largely side‐lined in pandemic responses as will be explored below.

### Children side‐lined in pandemic responses

Although in most cases children are less likely to become seriously ill with Covid‐19 compared to adults (Ludvigsson, 2020), this has contributed to governments marginalising children in their responses to the pandemic (Aitken, 2021; Bessell, 2021). However, the mental health impacts from the pandemic on children are likely to last for some time (Ford et al., 2021). Children's rights have been curbed more than those of adults such as their right to education, play, participation, association, assembly and a cultural life (Cortés‐Morales & Morales, 2021; Lundy et al., 2021). At times, children have been stigmatised in the media as ‘super spreaders’ of the virus responsible for the spread of Covid‐19 to more vulnerable members of a family or society (Lundy et al., 2021). This has resulted in stricter measures being placed upon children (Cortés‐Morales & Morales, 2021).

In the UK, children and young people have also been unhelpfully referred to as a ‘lost generation’ (National Association of Head Teachers [NAHT], 2021). When policy debate and media coverage has included the impact of lockdown on children, focus has largely been on their education and the need for them to ‘catch‐up’ on lost school days (Holt & Murray, 2021). However, even in this regard it seems that children have not been a priority in pandemic planning (Savage, 2021). For instance, the former Education Recovery Commissioner, Sir Kevan Collins resigned after the UK Government disregarded his proposed recovery fund for education of £15 billion opting to spend only £1.4 billion instead (Stewart & Clews, 2021). Similarly, whilst in Northern Ireland, Scotland and Wales, pledges were made to extend Free School Meals to children until the Easter of 2021, the vast majority of Conservative MPs once more displayed reluctance to do the same for children in England by voting down plans to extend this provision over the half term and Christmas holidays (UK Parliament, 2020). Again, this demonstrates how the devolved administrations diverged in their policy responses this time, regarding Free School Meal provisions. Arguably, these narratives of ‘catching up’ have also overshadowed other ways in which children's lives have been negatively impacted during the pandemic such as bereavement (Holt and Murray, 2020), their mental health and well‐being (McMullan, 2021), the rise in gender‐based violence (UN Women, , 2020), increased levels of food insecurity (Food Foundation, 2021b) and heightened risks of exploitation (Rafferty, 2020). Concerns of a ‘secondary pandemic’ of child abuse and neglect have also been voiced (Driscoll et al., 2021).

### Modern slavery and children in the UK

There is no agreed‐upon definition of modern slavery (Mende, 2019). ‘Modern slavery’ is used by some academics and modern‐day abolitionists (Bales, 2005; Miers, 2003) to define various forms of contemporary enslavement such as forced labour, child soldiering, organ harvesting, forced commercial sexual exploitation, forced and early marriage, servitude, debt bondage and trafficking. However, there have been calls by those who adopt a critical approach to modern slavery (Chuang, 2015; Dottridge, 2017) to end or limit the use of the term ‘modern slavery’ due to concerns that the discourse is entrenched in imperialism and over concerns that the term may trivialise the atrocities of the transatlantic slave trade (Dottridge, 2017). In the UK, modern slavery has been the umbrella term adopted in the UK Modern Slavery Act 2015 (MSA hereafter) and since this article engages with the anti‐slavery policy responses in the UK context during the pandemic, the term modern slavery (whilst acknowledging it is a contentious term) will be used. This article also uses the term ‘survivor’ to refer to individuals who have exited their situations of exploitation. However, this does not mean that a survivor may not be at risk of re‐entering a situation of exploitation again for instance, a survivor may be re‐trafficked.

Different policies and strategies relating to modern slavery have been adopted across the four nations of the UK. Whilst the MSA 2015 applies to England and Wales, certain provisions are adopted in Scotland and Northern Ireland including the Independent Anti‐Slavery Commissioner, transparency in supply chains provisions and maritime enforcement (Home Office, 2022). In Wales, support services for survivors of modern slavery such as housing are devolved (Home Office, 2022). The Human Trafficking and Exploitation (Scotland) Act 2015 applies in Scotland and the Human Trafficking and Exploitation (Criminal Justice and Support for Victims) Act (Northern Ireland) 2015 applies in Northern Ireland, both of which offer slightly different provisions in relation to survivor support through the National Referral Mechanism (NRM hereafter). For example, the Scottish government provides support through Migrant Help and Trafficking Awareness Raising Alliance (TARA) whereas in Northern Ireland support is provided by Migrant Help and Women's Aid rather than the Salvation Army as is the case in England and Wales (Home Office, 2022). Analysis of the three Acts indicates that although the UK Government boasts that the MSA is ‘world leading’, the Northern Ireland and Scotland Acts are more comprehensive when it concerns victim protection and are arguably more survivor‐focused (Brotherton, 2019).

Irrespective of the divergence of policies in the devolved nations, the exploitation of children is widespread across all four nations of the UK (Lundy et al., 2020). Notably, of the 2945 people referred to the Home Office via the National Referral Mechanism (NRM hereafter) or the Duty to Notify between 1 January and 31 March 2021, 1330 (45%) were children (Home Office, 2021). Criminal exploitation (which comes under the forced labour category) made up the majority (52%) of these claims. Cases of modern slavery involving children also include UASC. However, it is not stipulated by the National Crime Agency whether only the children who report exploitation get referred to the NRM or all UASC (Lundy et al., 2020). Therefore, UASC may be vulnerable as those who have been trafficked and/or as those who are unaccompanied and face a heightened risk of becoming trafficked or re‐trafficked. Despite the number of children with experience of exploitation in the UK, children have been largely side‐lined in UK anti‐slavery policies.

### Children side‐lined in anti‐slavery policies

Prior to the pandemic, the UK Government's anti‐slavery agenda and practices had been criticised for failing to prioritise children and consult their views despite having pledged to take into consideration the United Nations Convention on the Rights of the Child (UNCRC hereafter) when developing new policies (Gearon, 2019; Lundy et al., 2020). Rather than being underpinned by a children's rights‐based approach, child trafficking has been largely framed as a criminal justice and immigration issue (Gearon, 2015) as can be seen by the use of the four Ps (Pursue, Prevent, Protect and Prepare) paradigm which is derived from counter‐terrorism and organised crime strategies (see Home Office Modern Slavery Unit, 2019). The childhood of trafficked children especially UASC also has been questioned by authorities who have carried out x‐rays and checked children's dental records to determine the age of victims (Crawley, 2007). Pearce (2011) discusses how children's narratives and experiences of trafficking have been viewed with suspicion by practitioners as a result of a culture of disbelief and where there has been a preoccupation with the child's immigration status or age.

However, child trafficking has also come to be conceptualised as a child protection matter (Gearon, 2019). In the UK, Children Services have a statutory duty to safeguard and protect children who may have been trafficked through the MSA 2015, the Children Act 1989 and 2004, the Children (NI) Order 1995 and the Children (Scotland) Act 1995. This welfare approach depends on the effective coordination between Children's Services and first responders such as certain non‐governmental organisations (NGOs hereafter) and statutory authorities (Gearon, 2019). Nonetheless, research suggests that professionals who are likely to come into contact with child victims of exploitation may lack the training and skills to know about their rights and how best to support them. For instance, Such et al. (2019) highlight how whilst healthcare providers may be in the position to identify and support child victims of exploitation they may lack awareness about what constitutes modern slavery and what they are able to do if victims are identified.

Furthermore, tensions can arise between welfare and immigration approaches to child trafficking. Reports indicate that there have been occasions when immigration agencies have not made referrals to Children Services and have therefore failed to address the child protection issues of children who may have been trafficked (ATMG, 2010). NGOs and researchers have raised concerns that child victims of trafficking are often criminalised and refused asylum rather than being viewed as those who have been exploited by traffickers. This, according to McLaughlin (2018), has the potential to place these children at risks of additional exploitation or re‐trafficking.

Therefore, even before Covid‐19, the majority of the UK's anti‐slavery policies and practices had not been child‐centred but rather largely immigration‐led despite evidence that the promotion of children's rights would lead to better outcomes for children who have been exploited (Bovarnick, 2010).

## METHODS

### Design

This research was a mixed‐methods study and included various strands: a rapid evidence review of emerging grey literature, interviews with survivors of modern slavery, two dialogue events with stakeholders from the anti‐slavery sector in the UK and USA. Additionally, a web‐based social media analysis was carried out between January and September 2020 and an online survey of survivors was undertaken in December 2020 and January 2021 by 102 survivors of which 56 were based in the UK. To offer more tailored policy recommendations, this paper will focus on the findings and demographics of the UK participants only.

### Rapid evidence review

The aim of the evidence review was to offer a coherent and robust documentation of the recorded impacts and the potential risks facing survivors of modern slavery between 1 March and 31 October 2020. Rapid evidence reviews are recommended by the WHO as a suitable tool in crises to help guide health systems' responses in rapidly evolving situations (Tricco et al., 2017). Analysis of 106 relevant sources of grey literature during the first phase of the pandemic was undertaken, of which 40 had an exclusively UK focus. A second aim of this scoping review was to gain an insight into the risks facing individuals at an elevated risk of becoming exploited as a result of the pandemic. A third aim was to map and synthesise mitigation strategies that had been implemented for survivors at national, local and single organisation levels.

### Interviews with adult survivors

Sampling was purposive and survivors were invited to participate by one of our key project partners Survivor Alliance‐ a survivor‐led anti‐slavery organisation with members from across the world which aims to amplify the voices of survivors.

Survivor Alliance invited survivors to take part through their regular newsletter, social media platforms and one‐on‐one conversations. Survivors were not asked about the type of exploitation they had exited nor about how they had left their situations of exploitation. Nine survivors (six female and three male) took part in three interviews. The first in January 2021, second in April 2021 and the final in July 2021. Interviewees were aged 25–50 years old of which two identified as Albanian, three Black/Black British, one Caribbean, one Asian, one Black African and one White Welsh when asked about their ethnicity. Out of the nine participants, three were either UK citizens or had refugee status granted and six were seeking asylum.

### Dialogue event

In March 2021, 154 survivors and individuals from support organisations spanning the anti‐slavery sector in the UK gathered via an online forum hosted by one of the main project partners––the Human Trafficking Foundation. Notably, some attendees were both service providers and survivors. There was a presentation which gave an overview of some of the emerging findings at that point in the project to set the scene. Participants were asked to select and participate in a break‐out group which focused on one of the following three themes: adult survivors' health, well‐being and access to support services, legal support and immigration‐related issues, and risks to children and young people. These sessions were participatory enabling attendees to reflect on ongoing challenges facing survivors, to share examples of good practice in response to some of these challenges during the pandemic, and to explore ways of responding to ongoing challenges. Break‐out groups were facilitated by a member of the research team or an individual from one of the partner organisations. There was a guide that helped to structure the conversations. Notes were taken during the sessions and collated for a policy briefing (see The Rights Lab, 2021a). It is important to note that the compiled reflections were not necessarily representative of the entire sector but based upon participants' own experiences or observations. Audio‐recordings were not taken and therefore, accounts recorded were not verbatim. However, these thematically categorised discussion summaries were shared back to the participants to ensure we had fairly represented the views of participants.

### Data analysis

Survivor Alliance transcribed, coded and analysed the interview data. Thematic analysis was employed to code and elicit themes that emerged from the evidence review and also from the notes that were taken at the dialogue event. After initial codes were created and themes were identified, these were reviewed (Braun & Clarke, 2006) by the wider research team and key project partners.

### Strengths and limitations

The main strengths of this study include the way it was designed in consultation with survivors of modern slavery in the form of a Research Advisory Group and in collaboration with project partners working in the anti‐slavery sector. It also sought the views and experiences of survivors through interviews (and surveys). Limitations of the study lie in the lack of direct consultation with children. However, children were not consulted because the team worked with Survivor Alliance to voluntarily recruit research participants whose members are above 18 years old.

Nonetheless, whilst it was not an original aim of the project to assess the impact of the pandemic on children specifically, it became apparent that children were a population at heightened risk of exploitation and that those already in situations of exploitation and those whose parents were survivors had been particularly affected by the pandemic and government measures to halt the spread of the virus.

Therefore, the themes and narratives that have emerged in relation to the unequal impact of Covid‐19 on these three groups of children will be discussed in turn.

## FINDINGS

### Children of survivors

Drawing on the evidence review, interviews and discussions at the online forum, there are indications that the impact of lockdowns, school closures and the migration from in‐person to remote forms of support has been specifically experienced among the children of survivors especially in relation to their rights to food, education, play and development.

### Right to education

During interviews, survivors within the asylum system discussed how whilst they had been provided with devices by a support organisation, they had often lacked adequate financial support to afford data. In the evidence review, there were reports that during the initial phase of the pandemic some children of survivors living in Home Office National Asylum Support (NASS) accommodation did not have access to digital devices, books, craft resources or even a sofa to sit on (Jimenez et al., 2021: 15). Additionally, older children of survivors (aged 18–25) who were living in adult asylum or NRM accommodation were not considered eligible for internet packages or digital devices that had been allocated by the Department for Education in response to the pandemic (Jimenez et al., 2021: 17). Therefore, these young adults were unable to continue their remote learning. At the dialogue event, a survivor‐participant shared how supporting her children's learning from home had been especially challenging as all of her children had to share a single mobile phone to complete schoolwork. This led her to make multiple phone calls until a charity offered the loan of a laptop (The Rights Lab, 2021a). Different strands of this research suggest that government initiatives meant to supply children from lower‐income families with laptops were not effectively rolled out and some were left to actively seek out assistance from third sector organisations. Therefore, this research indicates that whilst the digital divide existed and digital inequalities were experienced by survivors before the pandemic (Malpass et al., 2022), school closures and the shift to remote and blended learning further unveiled the scale of digital inequalities experienced by children from lower‐income families in the UK. These findings offer some insight into how school closures of 6 months in March 2020 (and later in January 2021) may have further widened the attainment gap (Longfield, 2020) when it came to the children of survivors.

### Right to food

During the pandemic, Free School Meals were temporarily extended to include children with No Recourse to Public Funds (NRPF hereafter) (Department for Education, 2021). This response was significant since children from families with NRPF face greater risks of food insecurity (Food Foundation, 2021a). Those with NRPF include those seeking asylum and survivors who have yet to receive a Conclusive Grounds decision. Whilst separate, modern slavery and asylum processes often converge as previously discussed in relation to child trafficking approaches. Nonetheless, the evidence review included reports by service providers that survivors encountered delays in obtaining Free School Meal vouchers for their children. These delays occurred in situations where email access was required but not possible for survivors due to digital exclusion (Jimenez et al., 2021: 12). The evidence review also discussed how the reduced capacity of food banks and panic buying had hindered survivors' access to food during the first months of the crisis with reports of some refugee and asylum seeker women not being able to feed their children (Jimenez et al., 2021: 13). Research commissioned by the Food Foundation (2021b), found that 1.5 million of the children they surveyed (aged 8–17) reported that they had experienced food insecurity during the pandemic. Therefore, this research contributes to these broader findings by offering insight into how one group of children (those whose parents are survivors) experienced increased levels of food insecurity during the pandemic.

### Right to play and development

Although interviews with children were not conducted as part of this study, analysis of interviews with survivors who had caring responsibilities for children offer some insights into their perceptions of how their children were being negatively impacted by the lockdowns. An interview participant discussed how being housed in a small apartment without access to a green space had been particularly challenging for her and her children during the winter lockdown, she states:

I used to… take the children out maybe to the park… It's, like, 24/7 you are in the house. You can't go out with the kids and with the rain, the weather is very cold and there's nowhere else to go. The parks are closed now, so no place to go… I think the kids they're always inside most of the time, you know, they're not happy, it's like they are in a bubble, like, you're restricting them‐ we are restricting them in one place and they don't have that freedom which they had when we didn't have the Covid… the kids also need their own work to do, and they're at home 24/7… most of the time it's me and the kids so it‐ it was crowded (female survivor, UK).

Not only does this survivor describe the house as crowded and there being no place for her children to play, she also reflects on the potential impact that these restrictions are having on her children's well‐being describing them as ‘not happy’ and lacking freedom. She also voices concerns about their education when she discusses how they have their ‘own work to do’. Other interview participants also expressed concerns about how being unable to let their children socialise may have negatively affected their social and emotional development.

A female survivor who was interviewed described the dilemma she found herself in when weighing the risks and benefits of placing her daughter in nursery, she states:

I was planning to put her in nursery…but with the rate of how the new strain of Covid is spreading so fast, I'm even scared, I'm having anxiety and paranoid about even risking to put her in nursery at the moment. I also feel like… it does really get to her, being indoors constantly… I worry… and I feel like, right now, every age, even the kids and adults are feeling some kind of anxiety, some kind of mental health… and it's a bit more worrying because her age, they can't really express much… so I'm… more concerned about that, I'm concerned about how she's going to be able to socialise with other kids when things does get a little bit‐ much better (female survivor, UK).

It is helpful to bear in mind that survivors are a population who can be at heightened risk of contracting Covid‐19 and developing complications associated with it because of barriers to healthcare access, living in overcrowded accommodation and pre‐existing health conditions that may be connected to exploitation current or historic (Armitage & Nellums, 2020). Although it is not possible to infer from this extract the existence of any of these risk factors, it is worth considering that anxiety about placing a child in day‐care (and school) may be more elevated among survivor parents where these additional risks may be present. This survivor holds these fears about contracting Covid‐19 in tension with her concerns around the negative impact isolation may have on her young child's longer‐term emotional and social development referencing how the pandemic has adversely affected many people's mental health. Although it is often a right which receives little attention by policy‐makers, practitioners and legal scholars (Peleg, 2019), and does not seem to have been a primary consideration in pandemic policies, children's right to development was a preoccupation in this research for interview participants who had caring responsibilities for children. This reiterates research conducted by The Sutton Trust which noted that 52% of parents had noted that the social and emotional development of their younger children had been negatively impacted by lockdowns and the need to prioritise the early years in educational responses to Covid‐19 (NAHT, 2021).

Whilst the majority of children across the country will have been profoundly impacted by school closures and lockdown restrictions, children facing socioeconomic deprivation have been unequally affected. This paper offers insights into how the rights of children of survivors, particularly, may have been hindered in cases where they were already marginalised because they live in smaller or overcrowded accommodation, lack access to green spaces, experience digital inequalities and financial hardship.

### Child victims of exploitation

The findings from the evidence review and the dialogue event indicate that diminished face‐to‐face contact and the switch to remote safeguarding and legal provisions specifically affected UASC and victims of child criminal exploitation.

### Right to participation and representation in legal processes

Due to the Covid‐19 pandemic and efforts to contain the virus, court hearings moved online and video and audio links were used by children and young people in the justice system as part of the Coronavirus Bill (Department of Health and Social Care, 2020). UK Government documents in the evidence review stated that the Barnardo's‐run Independent Child Trafficking Guardians (ICTG hereafter) Service pivoted its sessional work online in March 2020 (Jimenez et al., 2021: 37). The aim of the ICTG Service is to support trafficked and potentially trafficked children and take their views into consideration on matters which affect them––a provision made under section 48 of the MSA 2015. Therefore, according to the Home Office Modern Slavery Unit, ICTG Direct Workers continued to support children and worked alongside safeguarding partners with regards to issues related to immigration, social care and criminal justice (Jimenez et al., 2021: 37). Whilst this service continued during the pandemic, albeit remotely, it seems according to NGO reports in the evidence review that delays in Conclusive Grounds decisions caused by the disruption of the pandemic had hindered access to support for victims of child criminal exploitation (Jimenez et al., 2021: 17). Similarly, during the dialogue event, a participant discussed how these young people had been left in limbo due to a backlog in court processes and the halting of referrals (The Rights Lab, 2021a).

In the evidence review, there were also reports that some UASC had not been involved in the process of compiling their witness statements with immigration solicitors (Jimenez et al., 2021: 17). These children were concerned that as a result they may have been misunderstood or misrepresented by those representing them and ultimately, the credibility of their stories undermined by the Home Office. Therefore, their right to not only a fair trial, but also to be able to effectively participate within court proceedings according to Article 30 of the UNCRC (United Nations General Assembly [UNGA], 1989) had been hindered. Additionally, their concerns about the reliability of their accounts echoes previous research that has highlighted a ‘culture of disbelief’ in relation to migrant children who have been trafficked (Bovarnick, 2010; Crawley, 2007; Gearon, 2019; Pearce, 2011).

### Right to safety

As previously discussed, child protection practices were disrupted as a result of diminished face‐to‐face contact between children and safeguarding professionals, partial or full closure of schools and youth services, as well as the redeployment of safeguarding partners (Driscoll et al., 2021). In this research, there were reports in the evidence review that newly arrived UASC had initial health assessments postponed and had been required to self‐isolate for 14 days unsupervised (Jimenez et al., 2021: 25). Whilst children may be less likely to develop complications from Covid‐19, this is not the case for all children and delayed health screening may have made these young people vulnerable to Covid‐19. Additionally, leaving UASC to quarantine alone may have placed them at greater risk of being targeted by traffickers since most potential victims of child trafficking go missing within the first 48 hours of going into care (Department for Education, 2014). Before the pandemic, NGOs and scholars had raised concerns about the number of trafficked or potentially trafficked children going missing whilst in the care of local authorities (Lundy et al., 2020; Every Child Protected Against Trafficking [ECPAT] UK, 2018). Therefore, there were indications that these government measures along with the reduction in in‐person support had impinged upon UASC's right to safety and may have placed them at additional risks of being trafficked or re‐trafficked.

As Ruiz‐Casares et al. (2017) state, in emergency situations, existing child protection concerns continue or can become exacerbated (as discussed above), and new child protection issues can arise in such crises including various forms of child exploitation.

### Children at heightened risk of exploitation

The evidence review revealed that concerns were raised at the start of the pandemic (April 2020) that the disruption to support services ordinarily delivered in‐person prior to the pandemic, would be used by perpetrators to exploit children usually receiving this support (Jimenez et al., 2021: 25). A UN report in the evidence review, noted an increase in demand for child sexual abuse material and trafficking for online sexual exploitation and The National Center for Missing and Exploited Children in the USA also discovered that traffickers had been in discussion over how to use the pandemic for the recruitment of unsupervised children into the production of sexual material (Jimenez et al., 2021: 25–26).

In the UK, a Government report discussed how there had been a rise in online sexual exploitation of children during the first 4 months of the pandemic (Jimenez et al., 2021: 25–26). Whilst the survey strand of this research will not have captured the views of children, when asked about risky contact during Covid‐19 in relation to delivering services in the sex industry, 4% of participants stated they knew children who had been contacted offline and 2% online (The Rights Lab, 2021b). At the dialogue event, there were reports that some forms of child exploitation had migrated online (The Rights Lab, 2021a). The latter reflects research on child victims of criminal exploitation during Covid‐19 that found young people faced heightened risks of exploitation via social media (Brewster et al., 2021). What follows is an overview of policy responses captured in the evidence review in relation to the risks facing children living in the UK of entering exploitation.

### Policy responses to children at risk of exploitation

Although children have been largely out of focus in pandemic responses, the evidence review indicated that the UK Government and notably, the Scottish Government had flagged potential risks to the safety of children as a result of school closures and lockdown measures. For instance, in April 2020, the UK Government pledged emergency funding of around £76 million for various charities working with marginalised children (Jimenez et al., 2021: 37). The Government announced that £34 million of the £750 million promised to the voluntary sector would be given to charities offering support services to children (Jimenez et al., 2021: 37). The Department for Education funded Barnardo's ‘See, Hear, Respond’ project which initially ran from June 2020 and was extended until March 2021 and sought to support children living in England who did not have social care involvement but were at risk during the pandemic including those at risk of exploitation (Bright, 2021). To ensure the continued work of organisations which provide services that help safeguard children impacted economically by the pandemic in England and Wales, £7.6 million was promised to Voluntary Community and Social Enterprise in June 2020 (Jimenez et al., 2021: 37). Although it is not possible to pinpoint how much of these resources were specifically directed towards child victims of exploitation, it is clear that efforts were being made to support children at risk of harm during the first 7 months of the pandemic.

Additionally, in April 2020, the Covid‐19 Vulnerable Children's Hub was established by the Home Office alongside other government departments to facilitate a coordinated approach when tackling risks of sexual exploitation and abuse, modern slavery, domestic abuse and criminal exploitation of children (Jimenez et al., 2021: 37). In Scotland, the Covid‐19 National Child Protection Guidance was established by the Scottish Government which sought to ensure vulnerable children which included trafficked or those at risk of being trafficked would have support and be protected during the Covid‐19 pandemic. Also in Scotland, a Covid‐19 Children and Families Collective Leadership Group was set up in May 2020 with the aim of evaluating work related to vulnerable children at local and national levels in Scotland. However, a report evaluating Scotland's Covid‐19 response has since unveiled that important data were missing on how children had been impacted by Covid‐19 such as those affected by digital exclusion and those who had accessed educational support during the pandemic (Tisdall et al., 2020).

Whilst these early responses, captured in the evidence review, demonstrate an awareness of some of the potential risks of exploitation facing children as a result of the pandemic, it seems that the UK Government's response centred on allocating funding to charities already working with children and the Home Office focused on improving coordination between law enforcement actors and NGOs. Alternatively, the Scottish government appears to have taken a more welfare approach by taking steps to ensure the safeguarding of at risk children. However, other sources in this evidence review also indicated that those already in exploitation such as victims of criminal exploitation, and those who may have just exited exploitation such as UASC were in need of greater protection during lockdowns. Furthermore, their rights along with the children of survivors were negatively impacted by government measures, especially the recommendation that safeguarding work, education, welfare entitlements and legal provisions be carried out remotely, without the matching support and provisions to enable them to do this.

## CONCLUSION AND RECOMMENDATIONS

Whilst the pandemic has exposed gaps in existing policies, practices and welfare provisions for children living in the UK such as food insecurity, the attainment gap and the digital divide, it has also prompted responses such as the extension of Free School Meals, the provision of digital devices, improved coordination in anti‐slavery efforts, allocation of funding to charities working with children and child protection mechanisms. However, according to this study, it seems that child victims of modern slavery faced barriers to these entitlements due to digital inequalities despite the introduction of some of these policies by the UK governments during the pandemic. Additionally, reduced face‐to‐face legal services seems to have hindered the rights of child victims of criminal exploitation and UASC to participation and fair representation in legal processes. There were indications that some UASC's right to safety had been overlooked as a result of being left unsupervised to self‐isolate at a time when they are at greatest risk of being exploited and going missing from care.

Whilst the full impact of the pandemic on the exploitation of children remains an incomplete picture, different strands of this research identified already marginalised children as a population at an elevated risk of online exploitation or re‐exploitation.

This paper concludes by offering some policy recommendations that may help to improve support for the children of survivors, child victims of exploitation and those at risk of entering exploitation.

1. The digital divide needs to be addressed to enable fair access to education and to mitigate risks of food insecurity for children from lower‐income families. Wi‐Fi, data packages and digital devices should be provided for survivors and internet provision should be available for those living in supported accommodation.
2. The early years must be invested in and prioritised in the government's educational recovery plan (NAHT, 2021).
3. The extension of Free School Meal entitlements for children from families with NRPF should become permanent.
4. Barriers to children's right to participation and fair representation in legal processes whether conducted remotely or in‐person need to be removed for child survivors and waiting times for referrals and Conclusive Grounds decisions need to be reduced.
5. Newly arrived UASC should be supervised by relevant safeguarding partners during their first weeks in the UK especially if required to self‐isolate to protect them from being trafficked or re‐trafficked.

Finally, a return to ‘normal’ will not necessarily adequately achieve the rights of marginalised children since inequalities existed before the outbreak of Covid‐19 as a result of a decade of austerity measures and welfare reforms. Similarly, anti‐slavery policies before the pandemic had been largely immigration‐based rather than child‐centred. As a result of the pandemic and it is unequal impact upon children with experience of exploitation, it is even more pertinent as Lundy et al. (2020) argue, that children and young people should have not only their protection rights, but also their participation rights upheld and their views given due weight in pandemic and anti‐slavery policies moving forward in accordance with Article 12 of the UNCRC. Therefore, the pandemic should be used as an opportunity to take stock, instigate structural change and invest in services that prioritise the rights of children in mental health care, education, free school meal provision, digital inclusion, legal processes but also enable their participation in anti‐slavery and pandemic policies as we learn to navigate the subsequent phases of the Covid‐19 pandemic.

## Data Availability

The data that support the findings of this study are available from the corresponding author upon reasonable request.
